# The NHV Rehabilitation Services Program Improves Long-Term Physical Functioning in Survivors of the 2008 Sichuan Earthquake: A Longitudinal Quasi Experiment

**DOI:** 10.1371/journal.pone.0053995

**Published:** 2013-01-07

**Authors:** Xia Zhang, Jan D. Reinhardt, James E. Gosney, Jianan Li

**Affiliations:** 1 The First Affiliated Hospital of Nanjing Medical University, Nanjing, People's Republic of China; 2 World Health Organization Liaison Sub–Committee on Rehabilitation Disaster Relief of the International Society of Physical and Rehabilitation Medicine, Geneva, Switzerland; 3 Swiss Paraplegic Research, Nottwil, Switzerland; 4 Department of Health Sciences and Health Policy, University of Lucerne, Lucerne, Switzerland; University of Pittsburgh, United States of America

## Abstract

**Background:**

Long-term disability following natural disasters significantly burdens survivors and the impacted society. Nevertheless, medical rehabilitation programming has been historically neglected in disaster relief planning. ‘NHV’ is a rehabilitation services program comprised of non–governmental organizations (NGOs) (N), local health departments (H), and professional rehabilitation volunteers (V) which aims to improve long-term physical functioning in survivors of the 2008 Sichuan earthquake. We aimed to evaluate the effectiveness of the NHV program.

**Methods/Findings:**

510 of 591 enrolled earthquake survivors participated in this longitudinal quasi-experimental study (86.3%). The early intervention group (NHV–E) consisted of 298 survivors who received institutional-based rehabilitation (IBR) followed by community-based rehabilitation (CBR); the late intervention group (NHV–L) was comprised of 101 survivors who began rehabilitation one year later. The control group of 111 earthquake survivors did not receive IBR/CBR. Physical functioning was assessed using the Barthel Index (BI). Data were analyzed with a mixed-effects Tobit regression model. Physical functioning was significantly increased in the NHV–E and NHV–L groups at follow-up but not in the control group after adjustment for gender, age, type of injury, and time to measurement. We found significant effects of both NHV (11.14, 95% CI 9.0–13.3) and sponaneaous recovery (5.03; 95% CI 1.73–8.34). The effect of NHV-E (11.3, 95% CI 9.0–13.7) was marginally greater than that of NHV-L (10.7, 95% CI 7.9–13.6). It could, however, not be determined whether specific IBR or CBR program components were effective since individual component exposures were not evaluated.

**Conclusion:**

Our analysis shows that the NHV improved the long-term physical functioning of Sichuan earthquake survivors with disabling injuries. The comprehensive rehabilitation program benefitted the individual and society, rehabilitation services in China, and international rehabilitation disaster relief planning. Similar IBR/CBR programs should therefore be considered for future large-scale rehabilitation disaster relief efforts.

## Introduction

Long-term physical disability following natural disasters significantly burdens the impacted society, even more so than immediate medical needs [1], [2], [3]. Nevertheless, medical rehabilitation programming has been historically neglected in disaster relief planning [4], [5], [6], [7], [8], [9]. Robust scientific evidence on effectiveness of medical rehabilitation in victims of earthquakes has not been previously reported in the medical literature [4]. The Sichuan earthquake of May 12, 2008 affected 46 million people, resulting in 87,476 deaths and over 350,000 persons injured, more than 10,000 of them severely [10], [11]. Many of these had disabling injuries, including fractures, amputation, spinal cord injury (SCI), and traumatic brain injury (TBI), all of which require physical rehabilitation to optimize long-term physical functioning and prevent medical complications [12], [13], [14], [15], [16].

The large volume of traumatic injuries overwhelmed the severely damaged local medical infrastructure of Sichuan province, resulting in mass evacuation of medically stable patients to hospitals across China [17], [18]. Anticipating the significant surgical and physical rehabilitation needs of the returning victims [19], [20], the Chinese Association of Rehabilitation Medicine (CARM) partnered with local health ministries as well as non-governmental organizations (NGOs) (N), local health departments (H), and rehabilitation volunteers (V) to form NHV, the medical relief strategy's rehabilitation services component. Local health department resources were capacitated by volunteer rehabilitation professional expertise and NGO funding and other resources in providing a comprehensive continuum of institutional and community–based rehabilitation (IBR, CBR) services designed to improve the long-term physical functioning and quality of life of injured survivors [7].

**Figure 1:**
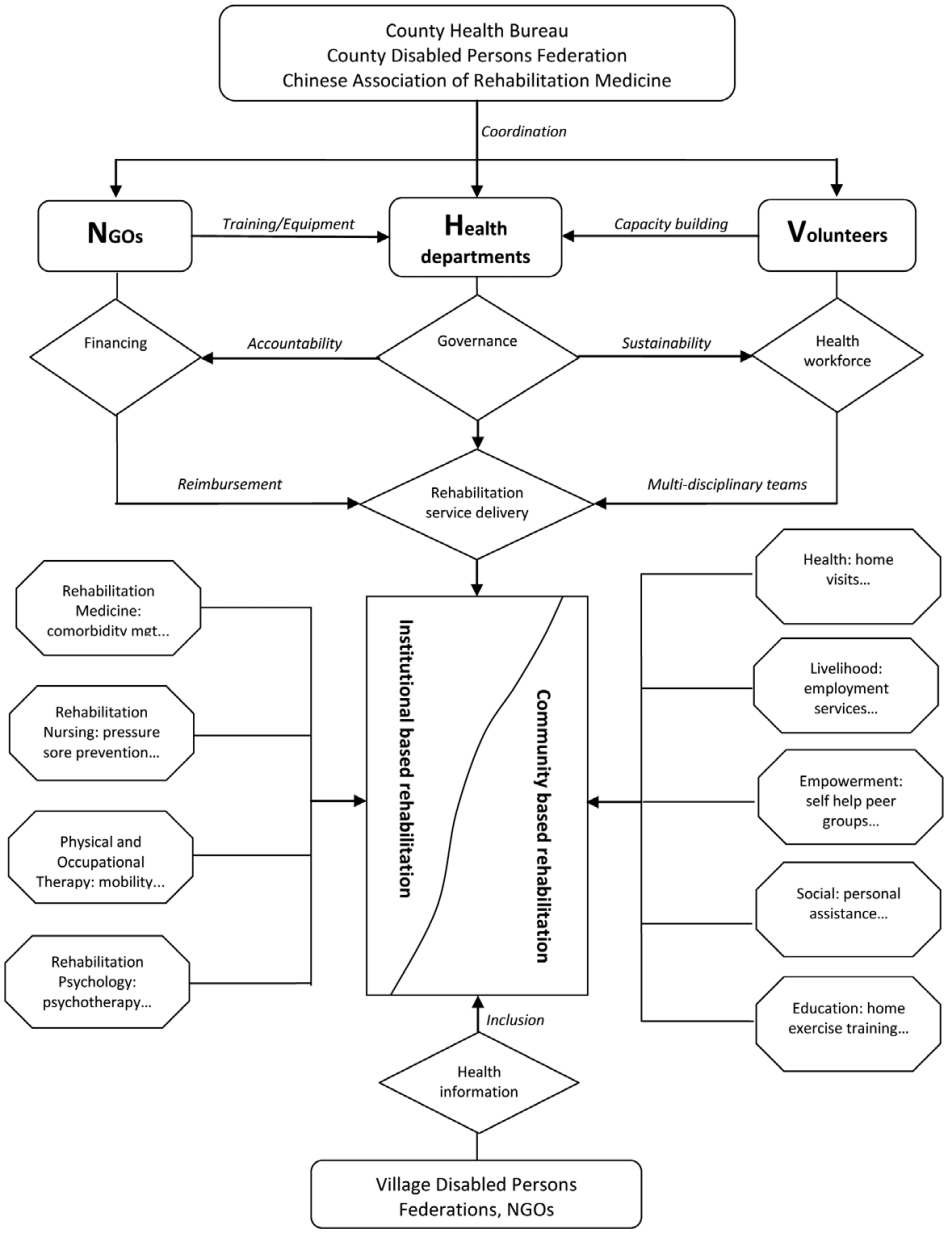
NHV program components and rehabilitation services.

IBR was administered in county hospital rehabilitation departments where patients participated in individualized physical rehabilitation programs. Rehabilitation interventions included muscle strengthening and range of motion exercises, training in self care and mobility activities, education in bladder, bowel and skin care management, and provision of assistive devices. Traditional Chinese therapies including acupuncture and massage were also provided. Following discharge to the community, CBR health sector services including medical care, rehabilitation, assistive devices, health prevention, and health promotion were provided [21]. Other CBR sectors comprising livelihood, social support, and empowerment were addressed via employment services, personal assistants, and patient self-help peer groups, respectively, among other interventions [21]. NHV focused initially on IBR and shifted to CBR as most earthquake victims were discharged into the community. Figure 1 shows the NHV program components and rehabilitation services.

Currently, NHV operates in two counties which accounted for more than 20% of the earthquake casualties [22]. A comprehensive prospective evaluation of NHV effectiveness attributing program causal effects has not been performed. Cumulative evidence on effectiveness of health service delivery following natural disasters is sparse due to compromised record keeping and lack of systematic planning [4], [6], [23], [24], [25]. Our study aims to quantify the effectiveness of the NHV rehabilitation program as measured by improvement in long-term physical functioning of Sichuan earthquake survivors with disabling injuries as assessed by the Barthel Index (BI).

## Methods

### Ethical Statement

This research has been approved by the ethical review board of the Medical Faculty of the Nanjing Medical University. Informed consent has been obtained from all study participants. All clinical investigations have been conducted according to the principles expressed in the Declaration of Helsinki.

### Design

We use a longitudinal quasi–experimental design with two points of measurement and two intervention groups and a control group.

### Setting

NHV IBR was implemented initially in a hospital in County A (NHV-E) which provided specialized rehabilitation care in its newly established rehabilitation department as part of comprehensive medical management of victims. CBR services were concurrently implemented in the community for patients' benefit following discharge by an international rehabilitation services NGO. Due to the urgent need to rehabilitate additional victims and given NHV's operational efficiency the program was implemented in neighbouring County B after one year (NHV-L). Due to resource and political constraints NHV was not implemented in neighbouring county C which served as the control group. Earthquake impact and victim demographics were comparable in the neighboring counties evaluated.

### Participants

**Figure 2:**
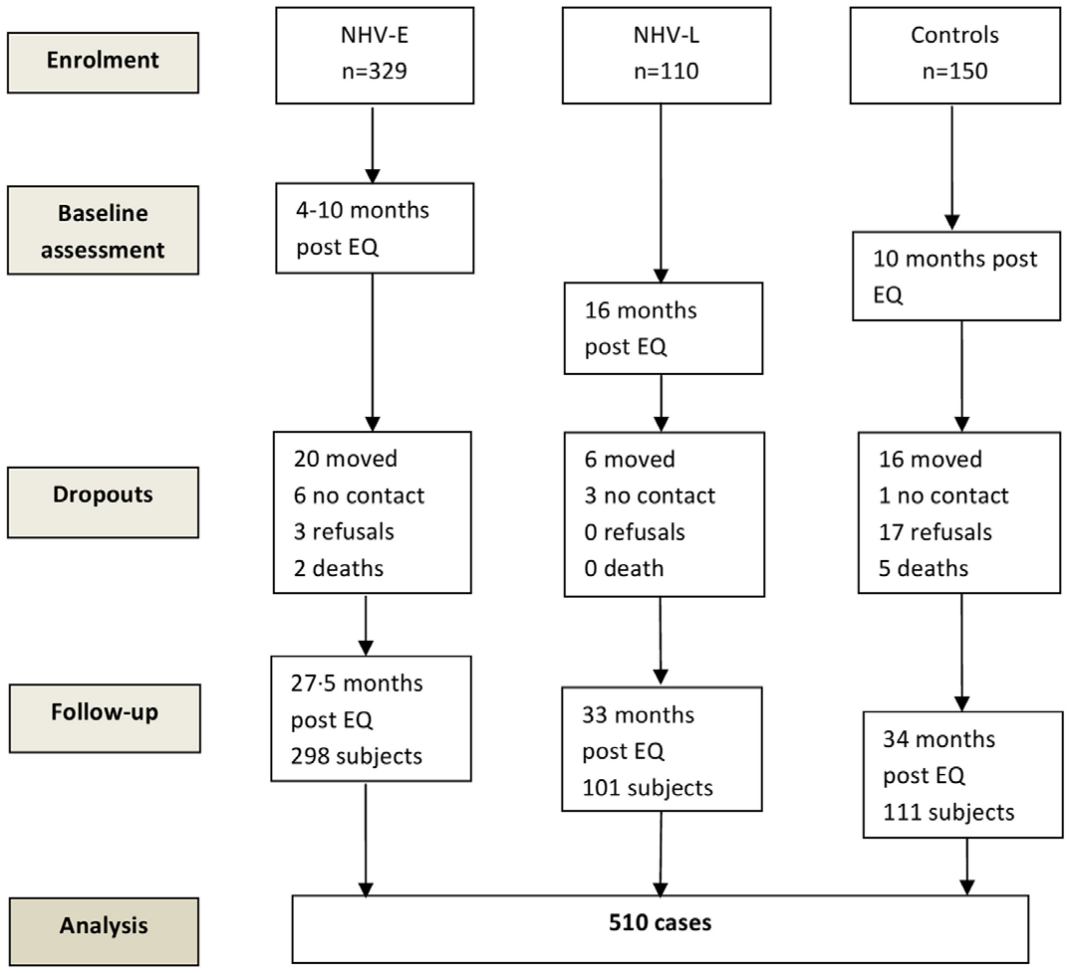
Subject flow in the NHV effectiveness study.

Five hundred and ninety-one earthquake survivors were originally identified and enrolled. Eligibility criteria for this study were: 1) age of 18 years and older; 2) diagnosis of a disabling injury requiring physical rehabilitation caused by the earthquake, and 3) no complicating internal injury. Based on these criteria, two patients were excluded due to complicating internal injuries. Written informed consent was obtained from participants in the intervention groups. Verbal telephonic consent was obtained from patients in the control group due primarily to limited volunteer availability. Identical consent forms were used. Five hundred and ten subjects completed the study of which 298 subjects were assigned to NHV–E, 101 participants to NHV-L, and 111 to the control group (Figure 2).

### Measures

Physical functioning was assessed with the Chinese version of the Barthel Index (BI), a measure of performance of ADLs, comprised of personal hygiene, bathing, eating, toileting, ambulation, dressing, bladder and bowel control, transfers, and mobility [26]. BI scores range from zero to 100 with higher scores indicating greater independence [26]. The BI was used since it enhances comparability with other Chinese data on rehabilitation of disabled persons as it is the most widely used non-injury specific assessment measure of functioning in China; Chinese versions of comparable measures used in international research have not yet been validated.

Diagnostic data on injury type were retrieved from patient hospital charts. Demographic information including gender and age as well as date of assessment were also recorded. Baseline intervention group data were obtained prior to the onset of IBR; NHV-E data were obtained at 4–10 months post-earthquake and data for the NHV-L group at 16 months. Baseline control group data were collected at 10 months post-earthquake. Follow-up data for the three groups were collected at 27.5 to 34 months (Figure 2). Measurement points were largely determined by the availability of volunteers for data collection, requiring mathematical adjustment for time to measurement (see below).

### Effect of NHV on daily physical functioning

**Figure 3:**
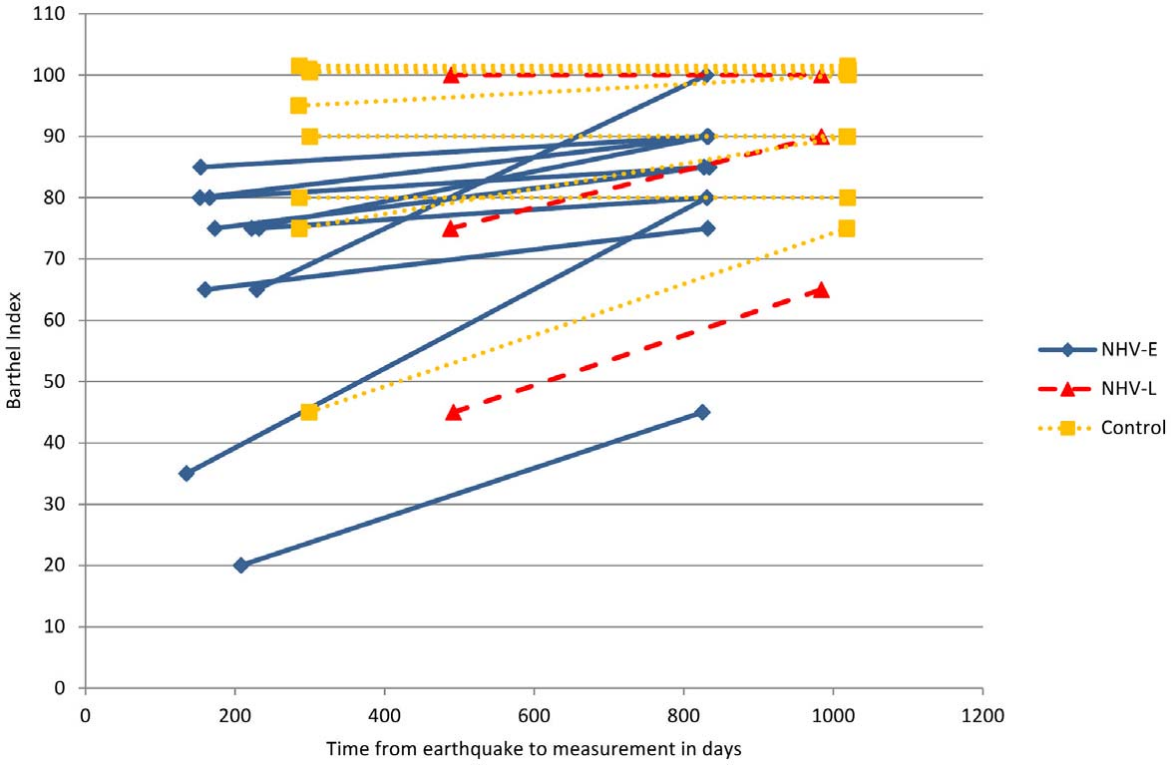
Ceiling effect of the Barthel Index in amputees (three patients from the control group and one from NHV-L begin with a censored score at baseline). Observations for one patient at different time points are connected by lines.

Due to a ceiling effect of the BI (Figure 3) we computed a longitudinal Tobit model to estimate the effectiveness of rehabilitation achieved through NHV-E and NHV-L [27], [28]. The use of this model in longitudinal analysis of BI data has been recommended by Twisk and Rijmen [24].

A ceiling effect implies that variance of BI scores cannot be measured above the scale maximum score due to the scale design. Scores, however, can be mathematically estimated with the Tobit model which employs a latent dependent variable that can assume values above the scale maximum. Estimation of supramaximal values allows correction for the baseline imbalance of physical functioning across study groups as the NHV-L and control groups had higher baseline values (see Table 1), leaving less margin for improvement up to the scale ceiling. The estimation of values above the scale maximum and the modelling of both an effect of time to measurement and of rehabilitation makes it possible to appropriately differentiate between influences of the rehabilitation program and the natural course of healing. Without correction for the BI's ceiling effect improvement in NHV-L and control group due to spontaneous recovery may have been underestimated.

**Table 1:** Baseline characteristics of the NHV study groups.

Characteristics	NHV–E (n = 298)	NHV–L(n = 101)	Control Group (n = 111)	P^‡^
Age, mean (SD)	55.2(16.6)	53.4(15.7)	51.8 (16.2)	0.156
Gender, % male	34.9	30.7	39.6	0.392
Injury classification, % (n)				
-Fracture	83.2% (248)	85.1% (86)	80.2% (89)	0.618
-Spinal cord injury	8.7% (26)	3.0% (3)	3.6% (4)	0.048
-Traumatic brain injury	1.7% (5)	1.0% (1)	6.3% (7)	0.017
-Amputation	3.7% (11)	3.0% (3)	7.2% (8)	0.226
-Other injury	2.7% (8)	7.9% (8)	2.7% (3)	0.045
BI at baseline, mean (SD)	77.5 (18.1)	80.1 (18.0)	82.4 (15.1)	0.032*
Time earthquake to baseline in days, mean (SD)	190.3 (32.5)	290.8 (9.1)	490.3 (1.5)	<0.001
Time earthquake to follow up in days, mean (SD)	829.5 (2.8)	984.4 (0.5)	1019.4 (1.1)	<0.001
Duration of Rehabilitation in days, mean (SD)	52.6 (19.1)	51.5 (19.0)	Not applicable	0.641†

SD = Standard Deviation; BI = Barthel Index. ^‡^F–test for metric; Chi–square for categorical variables. *Significant difference between NHV-E and Controls only, according to post hoc tests (Scheffé), ^†^T-test.

Since observations i (1,..,n_i_) at different time points (level 1) were nested in individuals j (1,…,n_j_) (level 2) who in turn were nested in different intervention groups (level 3) or counties k (1,2,3), random intercepts δ_j_ and φ_k_ were introduced on the individual and county level, respectively. To model individual responsiveness to recovery over time, a random slope γ_j_ for the time from the earthquake to the respective measurement points in days (TimeEQMP) was considered. In the fixed part of the model, BI values Y* (latent) were estimated using patients' age, TimeEQMP, and receipt of NHV rehabilitation services at follow up in the early (NHV_E_fu) or late intervention group (NHV_L_fu) as time-varying covariates. Gender (male = 1), injury type (dummies were introduced for fractures, amputation, SCI, and TB; other injuries was the reference group), and presence in an intervention (NHV_E_base vs. NHV_L_base) or control county (reference) were time-constant covariates. Random effects and residuals ε were assumed to be normally distributed with a mean of zero and variance of σ^2^.

The formula of the resulting three-level Tobit model is:
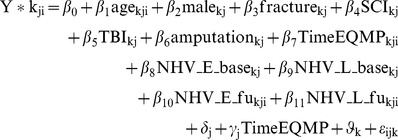
With



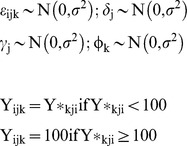



Based on the fixed parameter estimates of this model we predicted BI values for the study groups at baseline and follow-up while adjusting for other model covariates. Bonferroni correction was applied to adjust confidence intervals (CIs) for multiple testing.

### Counterfactual analysis

To differentiate the effect of IBR/CBR from the effect of recovery over time attributable to other factors, counterfactual predictions [29] were calculated by factoring out the rehabilitation effect in the model equation. Comparison of the slopes of change in BI from baseline to follow-up for the full and the counterfactual predictions indicates the difference between the NHV causal effect and the effect of recovery over time.

### Sensitivity Analysis

To determine the possible degree to which study dropouts may have resulted in estimation bias, the model was re-estimated by imputing BI dropout data for three scenarios: a) BI remained constant over time; b) BI decreased by ten points; c) BI remained constant in dropouts from the intervention groups while increasing by ten points in controls (censored observation stayed at 100). Fatalities were excluded.

All data were analysed with Stata 12. The three-level Tobit model was implemented with the gllamm command [27], [30]. All codes, estimates, analyses of residuals, and details of model development are available from the authors on request.

## Results

Table 1 shows baseline characteristics of the study groups. In terms of injury classification prevalence, significantly more patients are diagnosed with SCI in NHV–E, more patients with TBI are among controls, and more patients with other injuries can be found in NHV-L. Baseline BI scores and times from the earthquake to measurements vary significantly across groups, requiring statistical adjustment. Dropout characteristics are addressed with the related sensitivity analysis below. Without further adjustment, differences between baseline and follow up BI are significant in all groups (NHV-E: 13.6, 95%CI 11.3 to 16.0; NHV-L: 9.3, 95% CI 7.5 to 12.0; controls: 6.1, 95% CI 3.8 to 8.4).

Parameter estimates of the multi-level Tobit model (Table 2) show baseline physical functioning to be approximately 12 points lower in the NHV-E and 6 points lower in the NHV-L group than in controls. While NHV-E improved BI scores by about eleven points at follow up (11.3, 95% CI 9.0 to 13.7), the effect of NHV-L was marginally smaller (10.7, 95% CI 7.9 to 13.6). An effect of recovery over time is also noted as BI improved by almost one point over 100 days (0.0083*100). On the individual level, random intercept and random slope were strongly negatively correlated; the higher the baseline BI scores, the less the individual recovery over time.

**Table 2:** Parameter estimates for Barthel Index (BI) based on the three-level Tobit model.

Predictors	Coefficient	SE	z	95% Confidence Interval	
Age	−0.0328	0.0256	−1.3	−0.0831	0.0174
Male	1.3257	0.8428	1.6	−0.3262	2.9776
Fracture	6.8478	2.0357	3.4***	2.8579	10.8377
SCI	5.6715	2.571	2.2*	0.6325	10.7105
TBI	7.5046	2.9879	2.5*	1.6484	13.3607
Amputation	2.7665	2.8281	1.0	−2.7764	8.3095
Time from EQ to MP in days	0.0083	0.0016	5.2***	0.0052	0.0115
NHV-E baseline	−12.0477	1.51	−8.0***	−15.0073	−9.0881
NHV-L baseline	−5.9792	2.4302	−2.5*	−10.7424	−1.216
NHV-E follow up	11.3622	1.1982	9.5***	9.0137	13.7107
NHV-L follow up	10.7119	1.4589	7.3***	7.8526	13.5712
Intercept	79.2396	2.5876	30.62***	74.168	84.3112
*Variances/co-variances and SEs of random effects*					
Level 1	35.6958	3.4261			
Level 2 (subject): intercept	501.4740	28.8731			
Level 2 (subject): slope	0.0002	<0.0001			
Level 2 (subject): cov (intercept, slope)	−0.2609	0.0270			
Level 3 (group) intercept	<0.0001	<0.0001			

Log Likelihood: −3415.094; Bayesian Information Criterion (BIC): 6947.956.

SE = standard error, z = standardized coefficient; EQ = earthquake; MP = point of measurement; cov = covariance *p<0.05, **p<0.01, *** p<0.001.

**Figure 4:**
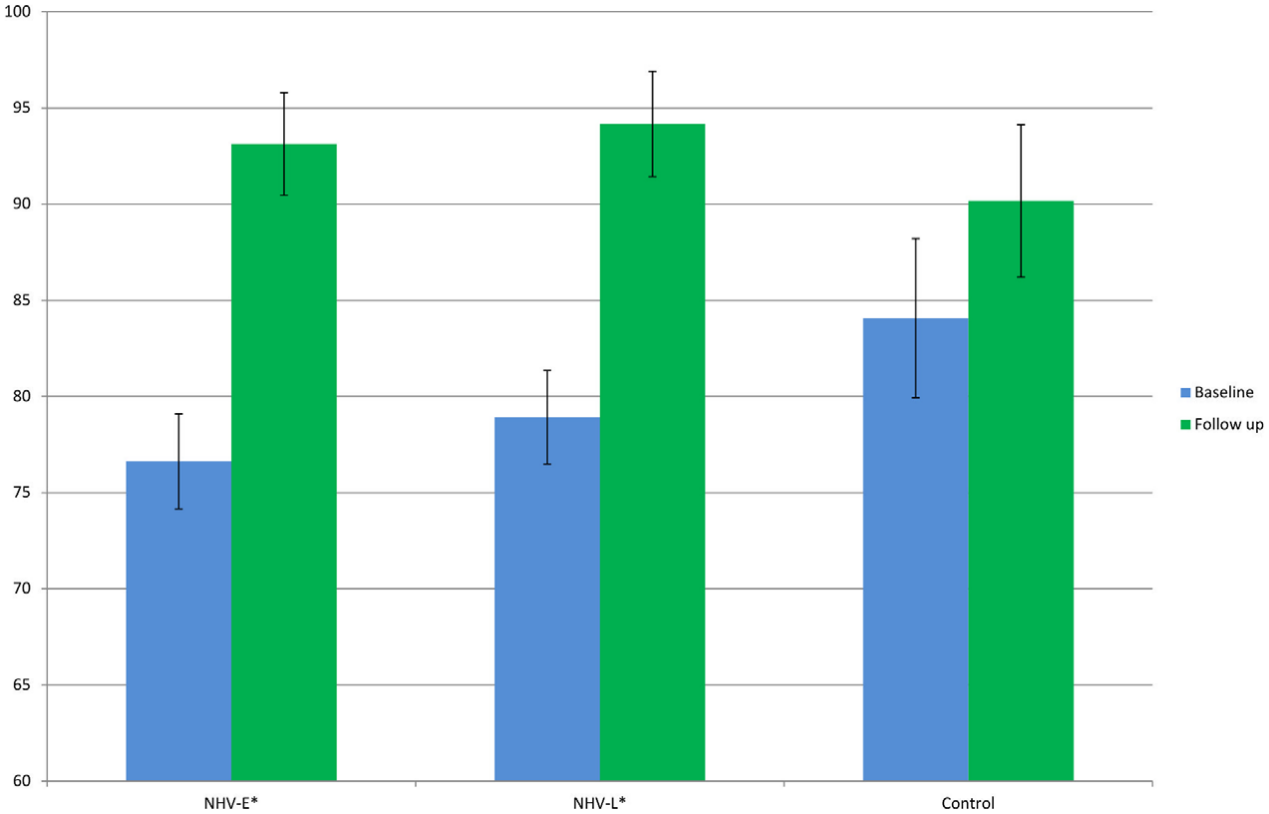
Marginal predictions of Barthel Index mean scores for the NHV study groups at time points based on the three-level Tobit model.

Marginal predictions show significant differences in baseline and follow-up BI scores in NHV-E (baseline: 73.8, 95% CI 72.5 to 75.2; follow up: 90.5, 95% CI 88.9 to 92.0) and NHV-L (baseline: 82.2, 95% CI 76.5 to 87.8; follow up: 96.9, 95% CI 92.4 to 101.4), while the difference is no longer significant in the control group (baseline: 86.8, 95% CI 83.6 to 90.1; follow up: 92.8, 95% CI 89.7 to 96.0) due to the absence of the NHV effect (Figure 4).

**Figure 5:**
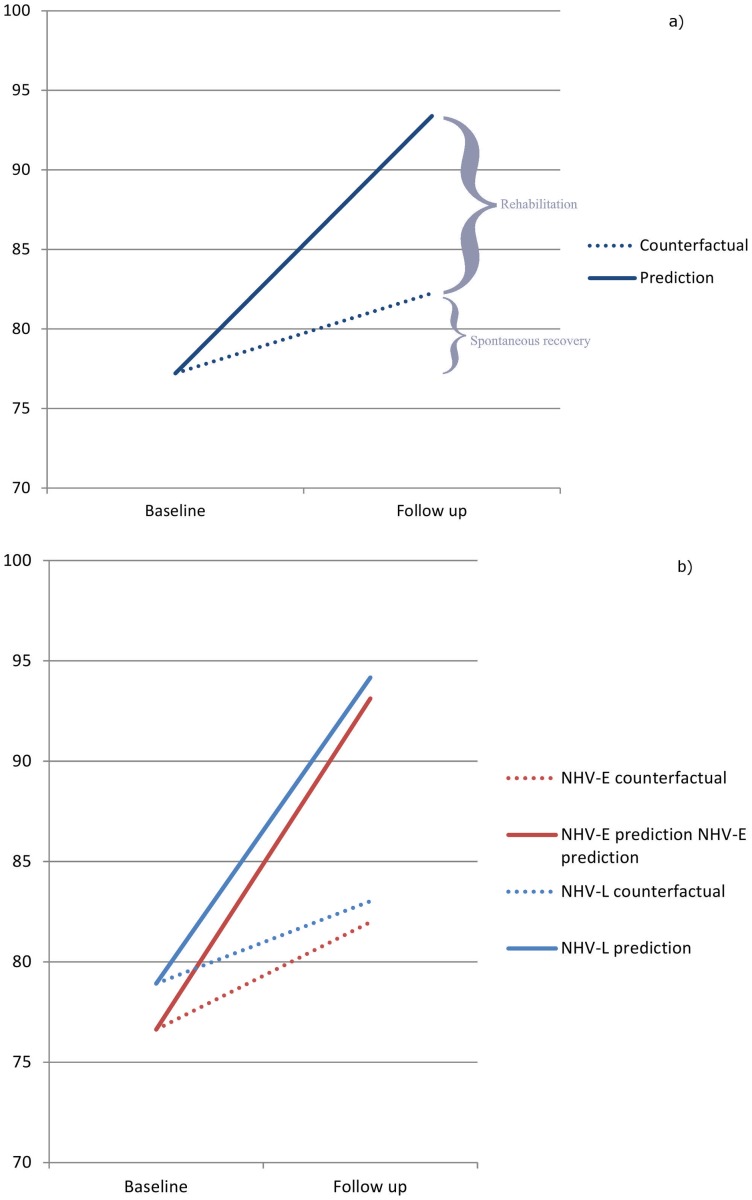
Relative combined effect of NHV and recovery over time (a) and individual effects of NHV-E and NHV-L and recovery over time (b).

Figure 5 shows the overall effect of NHV IBR/CBR (11.14, 95% CI 9.0 to 13.3) compared to recovery over time (5.03; 95% CI 1.73–8.34) (Figure 5a) as well as separate NHV-E and NHV-L group effects (Table 2) compared to respective recovery over time (Figure 5b). The positive effect on physical functioning due to NHV is nearly twice that of recovery over time. The recovery over time effect is greater in NHV-E than in NHV-L (5.3; 95% CI 2.7 to 7.8 vs. 4.0, 95% CI −2.1 to 10.1).

**Figure 6:**
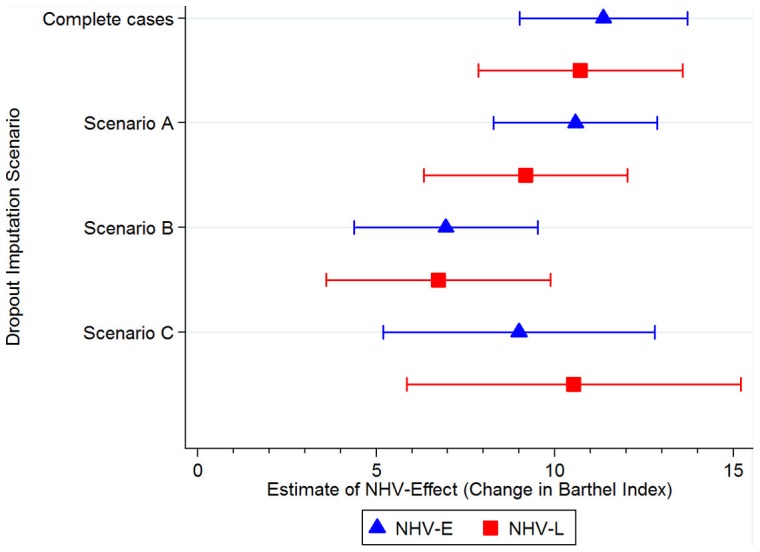
Sensitivity analysis: estimated change in NHV rehabilitation effect based on different dropout imputation scenarios. Legend: Scenario A  =  no dropouts incurred any change in Barthel Index; Scenario B  =  all dropouts decreased by ten points in Barthel Index at follow up; Scenario C  =  dropouts from the intervention groups did not change between baseline and follow up, while dropouts from the control group increased by ten points in Barthel Index; Complete cases  =  model estimation with valid cases only (see Table 2). Error bars represent 95% confidence intervals.

Regarding dropouts and their potential impact on estimation of NHV effects, more males dropped from the study than females (63.3 vs. 36.8%, Χ^2^ = 22.9, p<0.001). Dropouts were also significantly younger (38.2 vs. 54.1, T = 7.8; p<0.001) and had higher baseline BI scores in the intervention groups (94.3 vs. 78.1, T = 6.8, p<0.001). Sensitivity analysis showed the greatest relative decrease in NHV rehabilitation effects for imputation scenario B in which dropout BI scores were decreased by ten points (Figure 6). The NHV-E and NHV-L effects remained significant in all scenarios, however (p<0.01).

## Discussion

The NHV rehabilitation services program improved long-term physical functioning of severely disabled survivors of the 2008 Sichuan earthquake. Our fully adjusted multi-level Tobit model estimated a statistically significant and clinically meaningful 11 point increase in BI in the intervention groups compared to controls; patients who received comprehensive IBR/CBR in two heavily-impacted, neighboring counties (sequenced implementation) benefitted. Statistically meaningful improvement in physical functioning was thus shown in both early and late intervention groups, demonstrating benefit from rehabilitation delivered nearly 1.5 years after injury.

To compare our results with findings from the literature, we performed a systematic review (May 2012) in PubMed, Embase, and CINAHL using the search terms “rehabilitation” AND “earthquake” AND (“outcome”* OR” effectiveness”). Four studies reporting quantitative data on functional outcomes of rehabilitation interventions on adult earthquake subjects were identified; all investigated populations from Sichuan. Two employed a cross-sectional design with a control group [14], [31] and the other two used a pre-post intervention design [32]. A cross-sectional study on fracture victims of the Sichuan earthquakes revealed better outcomes in victims who received rehabilitation than controls at 27 months post-earthquake [14]. However, this study did not adequately adjust for baseline functioning. The other cross-sectional study was conducted in patients with tibial shaft fractures and performed a multivariate logistic regression to determine the effect of rehabilitation training on functional recovery. The odds of being in the two study groups which showed the greatest physical independence were five times higher for patients who received rehabilitation training [31]. However, this study is highly prone to bias since bivariate analysis was used to determine the multivariate model variables without correcting for multiple testing [33]. Li et al. [13] compared physical functioning at admission and discharge (institutional rehabilitation) in 51 Sichuan survivors with SCI and noted significant improvement. However, isolation of an effect of rehabilitation from spontaneous recovery was not possible since no control group was employed. Ning et. al [32] studied a team-based rehabilitation intervention and reported that the percentage of patients with complete functional dependence decreased from 89% at baseline to 38% at follow-up. However, it is unclear how functional dependence was measured, no control group was used, and no adjustment for other factors was performed.

Evidence on the effectiveness of medical rehabilitation in earthquake victims is scarce. Of the four relevant studies, two were of questionable methodological quality and none employed a pre-post intervention design using a control group. Our study is the first to employ a longitudinal design with a control group, allowing estimation of a causal effect of rehabilitation programming on physical functioning. The comparably large sample sizes allowed for appropriate adjustment for patient demographics and injury types. This study thereby contributes significantly to the evidence base on medical rehabilitation of earthquake victims.

Limitations of our research require that its results be viewed with caution, however. Counterfactual analysis allowed us to differentiate the effect of NHV IBR/CBR from that of recovery over time. However, although effectiveness of the NHV IBR/CBR program was demonstrated, it could not be determined whether specific IBR or CBR program components were effective since individual component exposures were not evaluated. IBR is believed to have made a significant contribution as has been found in other studies [13] since individualized therapy programs were designed to improve specific functional outcomes, including ADLs. However, BI was not administered at discharge, precluding isolation of an effect due to IBR. The effect of CBR would be more challenging to determine since discharged patients received various CBR health sector services, including rehabilitation (less structured and administered primarily by the patient or caregiver), in addition to other CBR sector services which likely positively affected functional outcomes. Future studies could be designed to distinguish between relative IBR and CBR effects as well as subcomponent effects on rehabilitation program effectiveness (the effect of specific IBR therapies or of CBR self-help peer groups, for example). CBR effects are expected to be further clarified by results of the third victim assessment performed in 2012 (data unavailable).

Although the BI ceiling effect was corrected using a Tobit model, actual improvement of patients who started IBR with censored scores was nonetheless not measured in this study. Future studies could employ other functional status measures which allow more precise evaluation of functional improvement, potentially based on WHO's International Classification of Functioning, Disability and Health (ICF) [34]. Measures of physical functioning that can be used for both clinical evaluation as well as population-based, survey assessment remain to be developed, however [4], [35].

Increased dropouts in controls compared to intervention groups may have also biased results; nonetheless, the NHV effect remained significant in our sensitivity analysis. Younger persons with better functional status at baseline dropped from the study, presumably migrating to other counties for employment [36]. Overestimation of the effect of recovery over time (population level) may have resulted since patients starting with higher baseline functioning are more likely to show smaller recovery over time effect based on random effect analysis.

Our study has significant implications for rehabilitation disaster-related research, for the study population, for the impacted area, and for rehabilitation disaster relief within China and internationally. Regarding disaster-related research, this analysis reinforces the significance of systematic needs and functional assessment data collection in support of long-term outcomes follow up studies. Such studies are required to improve the scientific base of disaster relief planning [25].

Sichuan earthquake victims with improved physical functioning are able to perform daily activities more independently, requiring less assistance. Moreover, they can perform household domestic tasks and work outside the home, contributing to the overall recovery of post-disaster society with reduced demand on health and social services. Greater victim community integration also enhances personal empowerment and well-being. The NHV rehabilitation services programme has thereby reduced the potential long-term burden of the earthquake on individuals and the community in this resource-constrained area.

The significant development of rehabilitation infrastructure via NHV demonstrates how large-scale natural disasters, particularly earthquakes, present an opportunity to expand rehabilitation services in low-resource regions. When the international rehabilitation services NGO departs in 2013 at the end of its five-year commitment, significantly developed IBR and CBR services will remain in place. Rehabilitation departments of hospitals in County A and B are now treating traumatic victims of motor vehicle accidents as well as other disabling injuries and rehabilitation diagnoses.

The rapid, close, and continued coordination between multiple rehabilitation stakeholders, including local authorities, national and international NGOs, and professional societies, serves as an example to guide future disaster responses in China and internationally. Capitalizing on the established operational mechanism and employing experienced program leadership, NHV was implemented in only half the time in County B (two weeks). The use of a professional volunteer recruitment database to hasten response underscores the recognized need within the international humanitarian response community for an international register of foreign medical team provider organizations [37]. NHV IBR/CBR also exemplifies the recognition within the international humanitarian community for close linkage of surgical and rehabilitation services as well as for a spectrum of required rehabilitation services following a large-scale disaster with significant disabling injuries [4], [9], [38].

In conclusion, our study provides evidence that the NHV program was effective in improving physical functioning of injured earthquake survivors of the 2008 Sichuan earthquake. The comprehensive rehabilitation program benefitted the individual and society, rehabilitation services in China, and international rehabilitation disaster relief planning. Similar IBR/CBR programs should therefore be considered for future large-scale rehabilitation disaster relief efforts.
